# Shape Controlled Synthesis of Copper Vanadate Platelet Nanostructures, Their Optical Band Edges, and Solar-Driven Water Splitting Properties

**DOI:** 10.1038/s41598-017-14111-7

**Published:** 2017-10-30

**Authors:** Ibrahim Khan, Ahsanulhaq Qurashi

**Affiliations:** 1 Center of Research Excellence in Nanotechnology, King Fahd University of Petroleum and Minerals, Dhahran, 31261, Saudi Arabia; 2 Chemistry Department, King Fahd University of Petroleum and Minerals, Dhahran, 31261, Saudi Arabia

## Abstract

We report the morphological and size tailored rational and facile synthesis of copper vanadate nanostructures via sonication assisted sol gel method. Field emission scanning electron microscopy (FESEM), indicated irregular and nanoflakes morphologies for the as synthesized copper vanadate (CV-120) and copper vanadate calcined at 250 °C (CV-250). The semispherical platelets shaped morphology revealed for the copper vanadate calcined at 550 °C (CV-500). The XRD patterns confirm the monoclinic and triclinic crystal phases for CV-250 and CV-500, respectively. The optical properties of CV-250 and CV-500 via UV-DRS showed significant absorption in the visible regime at λ = 565 nm and 670 nm with band gap 2.2 eV and 1.84 eV, respectively as calculated from Kubelka-Munk (KM) equation via Tauc’s plot. The values of band edge positions of CV-250 and CV-550 straddle with the hydrogen (HER) and oxygen evolution reaction (OER) potentials. The photoelectrodes of CV-250 and CV-500 fabricated by adsorption desorption method to test their photoelectrochemical (PEC) water splitting performance in the three-electrode cell. The onset photocurrent potential is observed at ~0.42 V, which reached to saturation at 1.05 V. The photocurrent density at saturation is ~0.65 mA/cm^2^ for CV-250 and CV-500, respectively.

## Introduction

Photoelectrochemical (PEC) conversion of solar energy into chemical forms is a major scientific challenge and is extensively pursued in research due to its promising environmental energy sustainability. Owing to exceptional optical properties and ease in synthesis, numerous metal oxide semiconductors such as TiO_2_^1–3^, Cu_2_O^4^, Fe_2_O_3_^5^, ZnO^6^, WO_3_^7^, and BiVO_4_^8^ etc. have been investigated as photoelectrode materials for solar-driven PEC water splitting. Most of these semiconductor nanomaterials have wide band gaps in their pristine form and therefore show limited PEC performance. The required band gap energy of the photoelectrode must be in the range 1.5–2.5 eV, where they absorb the maximum amount of visible-light. In addition to band gap, the photoelectrode should produce a large number of photogenerated charge carriers i.e. electron (e^−^) and hole (h^+^) pairs, show high chemical stability, have high corrosion resistance, and cost competitive^9^. Furthermore, the band edge positions of their conduction (CB) and valence bands (VB) should be such that they aid the absorption of as much visible light as possible^10^. Approximately, 42–43% of the sunlight that reaches earth lies in the visible range i.e. 420 < λ < 700 nm. Hence, photoelectrodes capable of absorbing radiation in the visible-light region are of primary interest to the PEC research community^3^. Being a p-type semiconductor oxide and having bandgap 2.0–2.2 eV, Cuprous oxide (Cu_2_O) can be a good substitute to absorb the maximum visible radiations of the solar spectrum. However, it is fragile and susceptible to photocorrosion due to intense photoexcitons recombination. Thus, the fragile stability of Cu_2_O has been one of the major drawbacks. In addition, the e^−^/h^+^ pairs offer much dragging forces by Cu_2_O and hence their flow rate decrease considerably^11,12^. Correspondingly, WO_3_^7^ and hematite^5^ photoanodes are also substantially photoactive in the visible. Nevertheless, these materials have susceptibilities towards short carrier diffusion length, inefficient charge separation, and significant recombination rate. Various approaches have been adopted to overcome these issues, which include doping of metals^13^ and nonmetals^14^. Defects and oxygen vacancies creation^15^, catalysis and co-catalysis^16^, using the surface plasmon resonance (SPR) of noble metals such as gold and silver^17^, composite and heterostructure formation of two or more photoactive materials^6,7,18^. Various composites and heterostructure oxides have been employed, such as RGO/ZnO^6^, Fe_2_O_3_/TiO_2_^19^, MoS_2_/graphene^20^ etc. with much-improved photocurrent densities and solar efficiencies. Metal vanadates (Mx(VO_4_)y), is emerging a new class of photoelectrode material for PEC water splitting. These are ternary oxides, which are composed of three atoms with varied elemental ratios. Among Mx(VO_4_)y, pristine BiVO_4_ and their hybrid have been investigated by various researchers due to their fascinating optoelectrical characteristics with a band gap ~2.40 eV^8,21^. Lately, copper vanadate has been explored for lithium-ion batteries^22,23^, dye degradation^24^, and water splitting applications^18,25^ due to their excellent optical and electrical properties, as well. It is an *n*-type photoactive material with the indirect band gap of ~2 eV.

Different forms of copper vanadate have been investigated with different stoichiometries, e.g. CuV_2_O_6_, Cu_2_V_2_O_7_, and Cu_3_V_2_O_8_^25,26^. The stoichiometric ratio alters the physicochemical behavior of these materials and hence the water splitting performance greatly varied for each material. In addition, morphology and particle size further effect the overall current densities of these materials. Very thin films of CuV_2_O_6_ and Cu_2_V_2_O_7_ were fabricated via a drop-casting method^8^. Both materials showed photocurrent response of 25 and 35 μA/cm^2^ at 1.23 V vs RHE in sodium borate (0.1 M) electrolyte, under visible irradiations. The same group also reported that CuV_2_O_6_ displays 2 folds higher photocurrent density than Cu_2_V_2_O_7_ at 1.58 V. Certainly, the overall photocurrent density values are at the lower side due to obvious reasons. Similarly, Seabold *et al*.^27^ prepared a Cu_3_V_2_O_8_, by vigorous shaking and centrifugation technique and used dip coating to fabricate the film over FTO electrode to see their performance for oxygen evolution reaction (OER). The photocurrent density was lower even after molybdenum (Mo) doping. More recently, Kim *et al*.^18^ engineered CuV_2_O_6_ and Cu_2_V_2_O_7_ films via electro-spraying method and report significant photocurrent density of 0.65 mA/cm^2^ for the later in the presence of Na_2_SO_3_ hole scavenger. Some detail comparison of the photocurrent generated from the different forms of copper vanadate is given in Table 1, with morphology and synthesis method.

**Table 1:** Reported photocurrent densities for copper vanadate nanostructures synthesized using different methods and with different morphologies.

**Copper vanadate Types**	**Method**	**Nanoparticles Morphology**	**Onset potential**	**Photocurrent at 1.23 V**	**Ref**.
CuV_2_O_6_	drop-casting method	Interconnected irregular shaped NPs	0.80 V	25 μA/cm^2^	25
Cu_2_V_2_O_7_	drop-casting method	Interconnected irregular shaped NPs	0.75 V	12 μA/cm^2^	25
Cu_3_V_2_O_8_	vigorous shaking and centrifugation followed by dip coating	Irregular shaped NPs	0.70 V	28 μA/cm^2^	27
**CuV**_**2**_ **O**_**6**_	**Pulse sonication assisted Sol-gel method**	**Uniform platelets shaped NPs**	**0.42 V**	**65–70** **μA/cm** ^2^	**This work**

**Figure 1:**
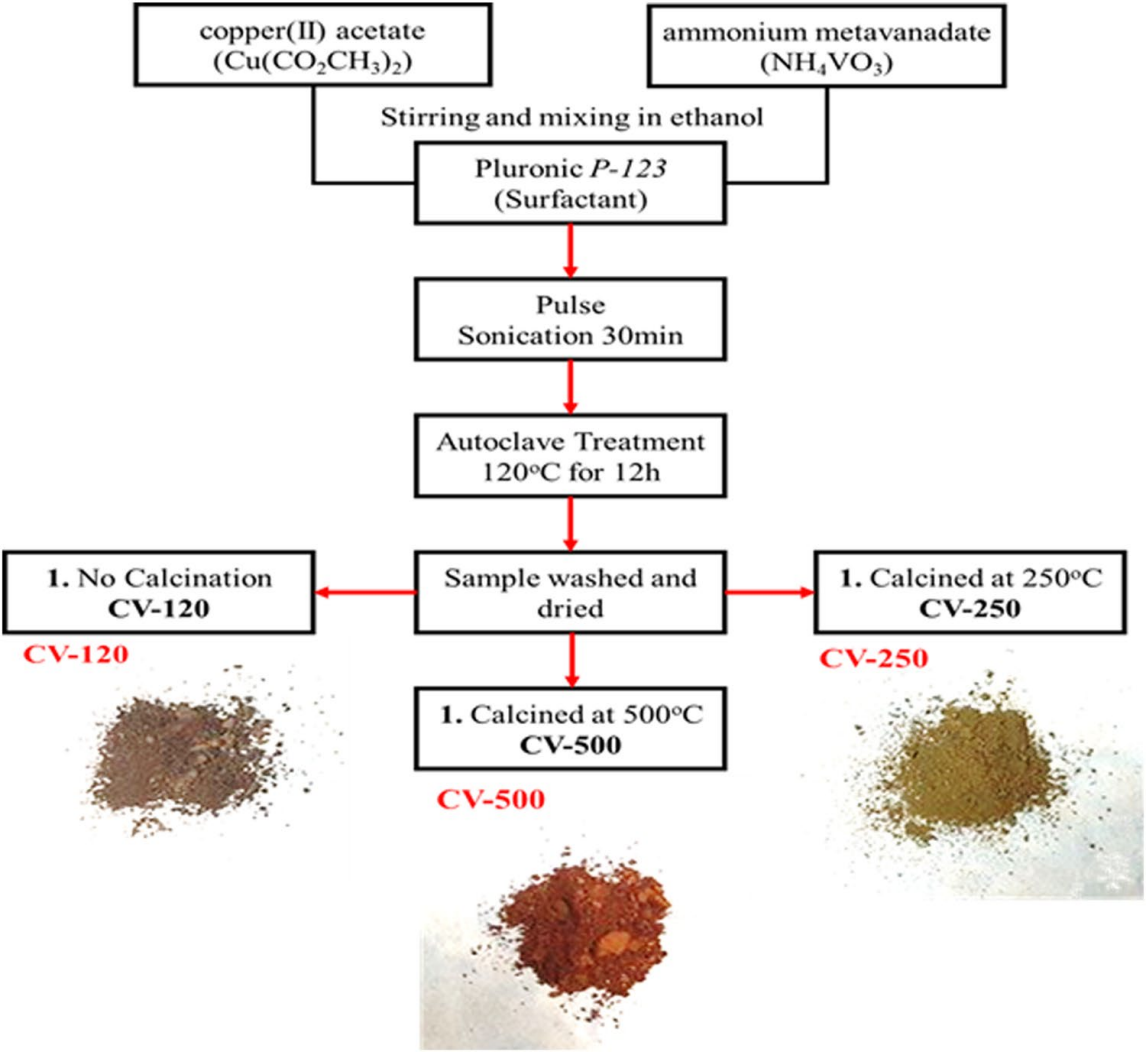
A scheme showing the preparation of different copper vanadate samples via sol-gel method.

As stated earlier, that in addition to other factors morphology and size control also enhance the PEC performance of the photoelectrode material to a certain extent and therefore the synthetic route and precursors are always pivotal to control these parameters. The aforementioned copper vanadates have been synthesized using hydrothermal, sputtering, solid-state synthesis methods with no data provided for the morphology and particle size effect on the water splitting performance. However, Herein, for the first time, we used pulse sonicated assisted sol gel method to produce CV-120, CV-250, and CV-500 photoelectrodes. Pulse sonication is performed to mix the precursors extensively with the surfactant (*P-123*) to achieve more uniform micelles, after which sol gel method is adopted for shape tailored growth of copper vanadates. The proposed growth mechanism with drawing (Fig. 1) is also provided.

## Results and Discussion

**Figure 2:**
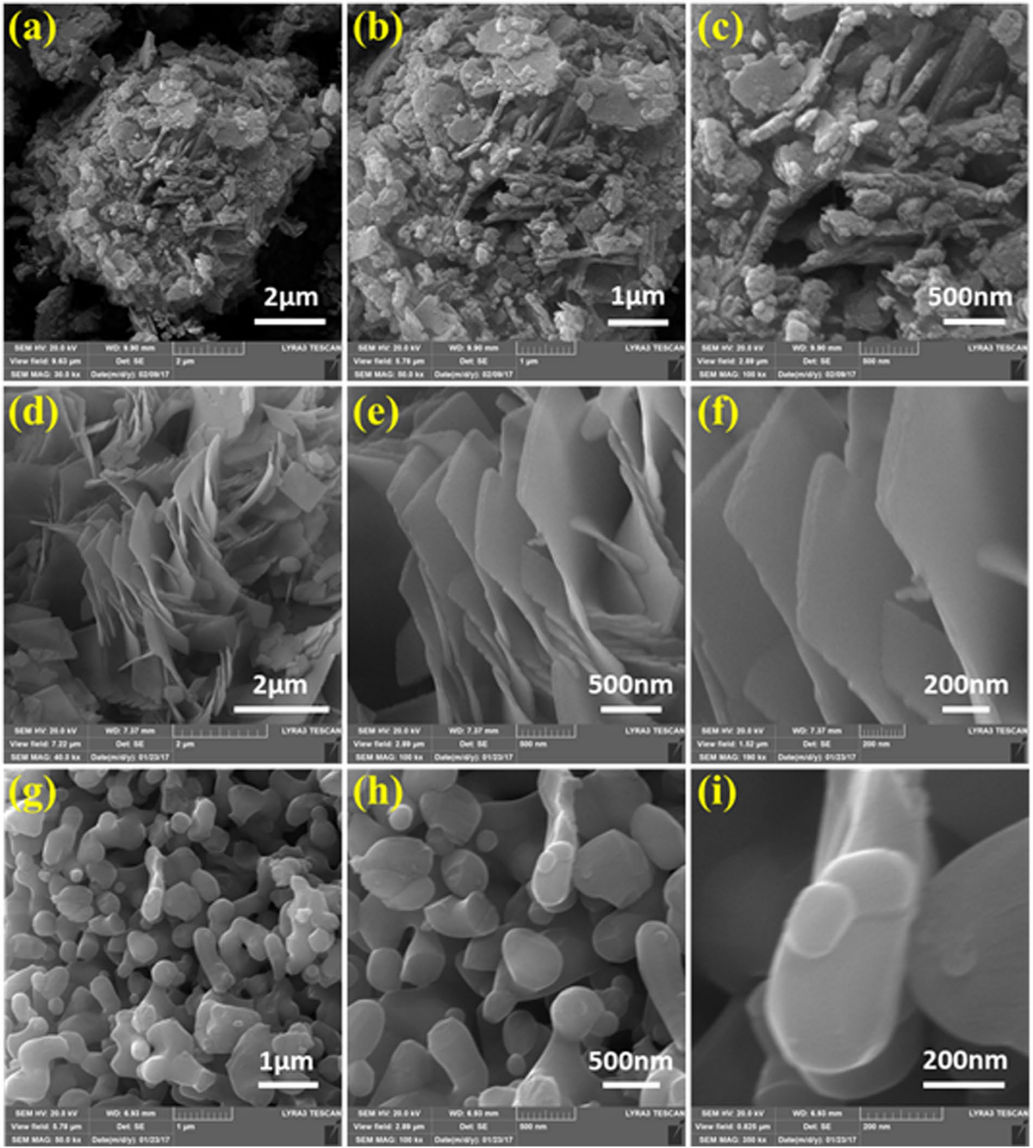
Different resolution SEM images of CV-120 (**a**–**c**), CV-250 (**d**–**e**) and CV-500 (**g**–**i**).

The morphological features of the samples explored through FESEM and the micrographs taken at different resolutions. Figure 2 revealed that morphological transformations take place with increasing the calcination temperature. The CV-120 sample showed variable morphological topologies with no significant features. The shapes of the nanoparticles are indistinguishable in this case as indicated by Fig. 2(a–c). The evolution of regular morphology can be seen in CV-250, after calcination at a higher temperature, where the micrographs show (Fig. 2d,e) sand-flower shaped textures with larger sizes. These microflakes arranged in channels pattern. Substantial transformation is observed in the case of CV-500, where the particle size is significantly decreased until 50–70 nm with peculiar platelets shaped appearance. The CV-500 platelets NPs are well dispersed and regular shape as shown in Fig. 2 (g–i). CV-120 is not investigated further, as it has insignificant morphological features.

**Figure 3:**
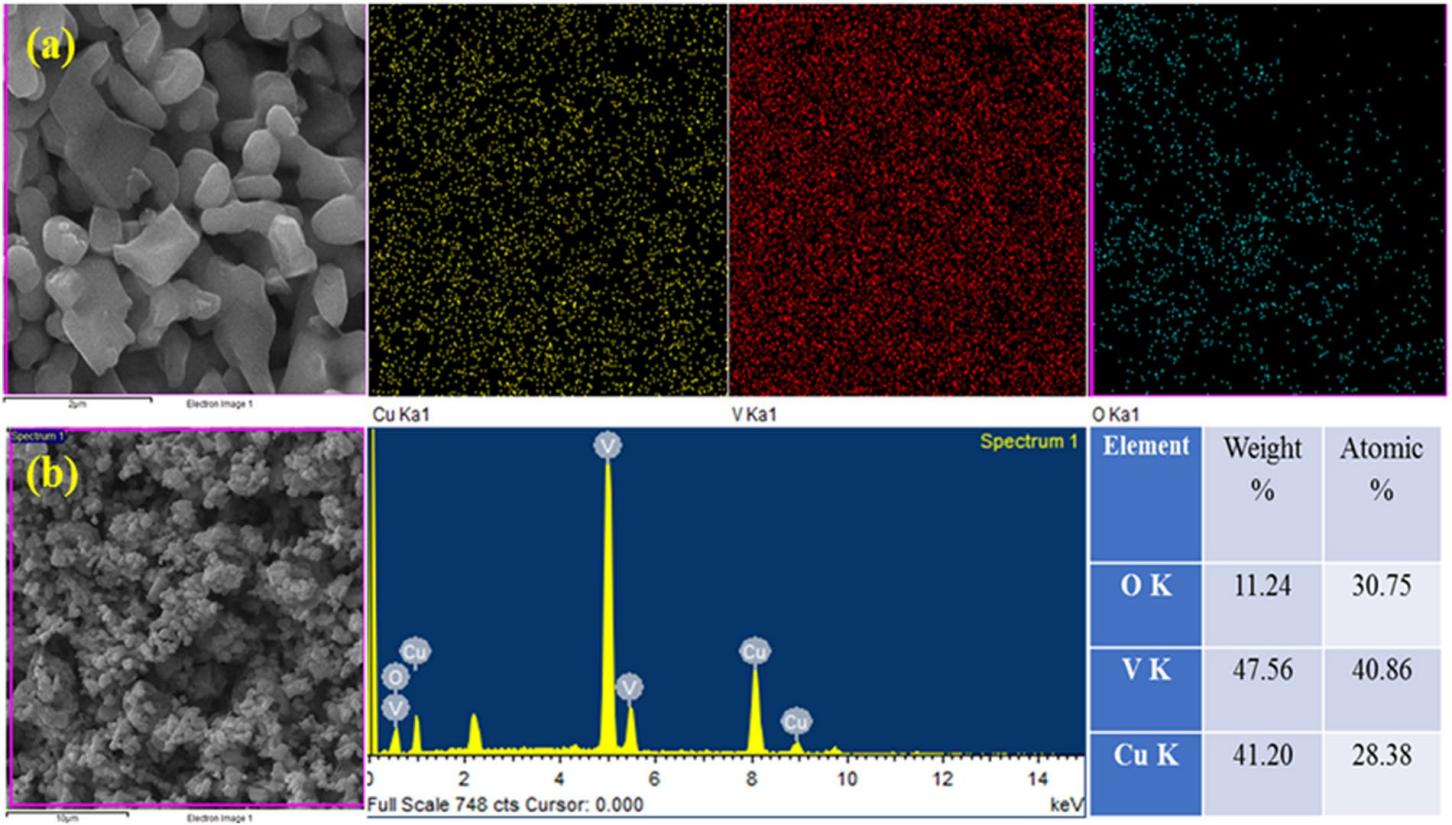
(**a**) Elemental mapping, and (**b**) EDX analysis of CV-550 nanoparticles.

Owing to stimulating morphological features, the elemental composition and their thorough dispersion in CV-500 sample further investigated via EDX and elemental mapping techniques. The elemental maps provided in Fig. 3(a), which is clearly indicating the dispersion of all constituent metal particles i.e. Cu, V and O in the CV-550 sample. Furthermore, the EDX in Fig. 3(b) also shows characteristic peaks arise from the inner K shells of respective elements. The % atomic-ratio for Cu, V and O are 28.38, 40.86, 30.75, correspondingly.

**Figure 4:**
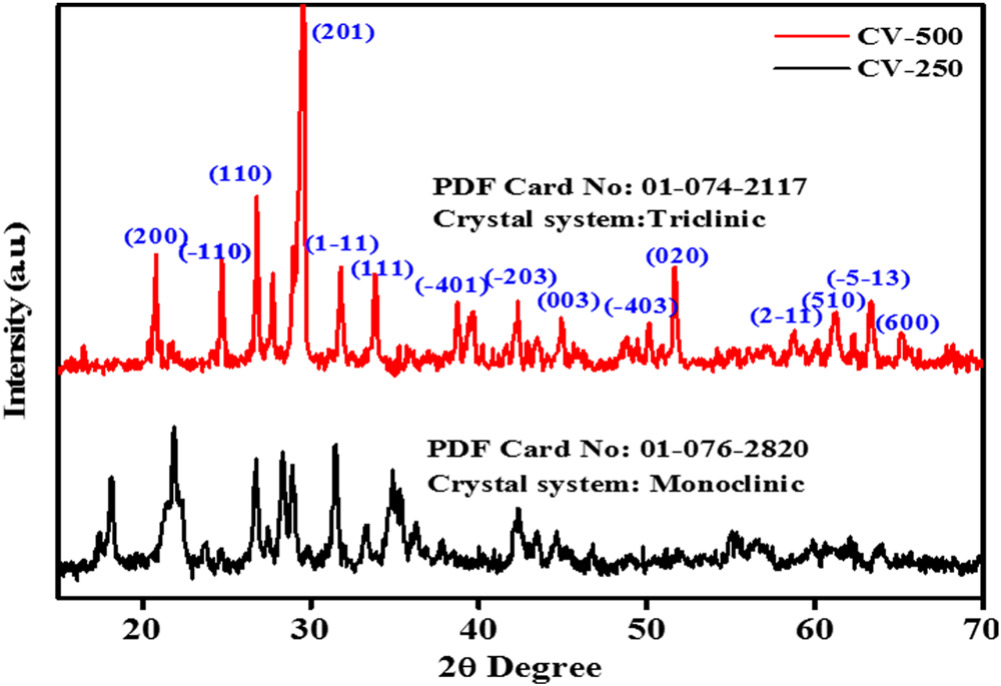
XRD Patterns of monoclinic CV-250 and triclinic CV-500.

The XRD patterns for CV-250 and CV-500 are given in Fig. 4. The XRD pattern of CV-250 is matching with the monoclinic, Cu_2_V_2_O_7_, with a space group C2/c (powder diffraction file (PDF) Card No: 00–076–2820)^28^. The unit cell parameters observed for CV-250 are: a = 7.69 Å, b = 8.03 Å and c = 10.11 Å, with α = 90°, β = 110° and γ = 90°. The total volume of the unit cell was 585.35 Å. Similarly, the CV-500 shows characteristic XRD pattern of triclinic CuV_2_O_6_ with a space group C-1 (PDF Card No: 01–074–2117)^29^. All the peaks are indexed for CV-500. The major XRD patterns are centered at angle (2θ) 20° (200), 26° (−100), 28° (110), 29° (201), 32° (1–11), 34° (111), 39° (−401), 42° (−203), 45° (003), 50° (−403), 52° (020), 58° (2–11), 60° (510), 63° (−5–13) and 65° (600), approximately. The unit cell parameters observed for CV-500 are: a = 9.17 Å, b = 3.54 Å and c = 6.48 Å, with α = 92.25°, β = 110.34° and γ = 91.88°. The total volume of the unit cell was 196.91 Å. The XRD patterns of CV-250 and CV-500 are sharp and intense, which can be related with a high crystallinity of the formed products. Moreover, average the particle size of CV-250 and CV-500 is determined from XRD patterns by using Scherer’s equation^8^. The average particle size for sand-flower shaped CV-250 is ~65 nm, and ~50 nm for platelets shaped CV-500 NPs. Further details about the unit cell parameters, d-spacing values, and particle size distribution are given in Table S1.

**Figure 5:**
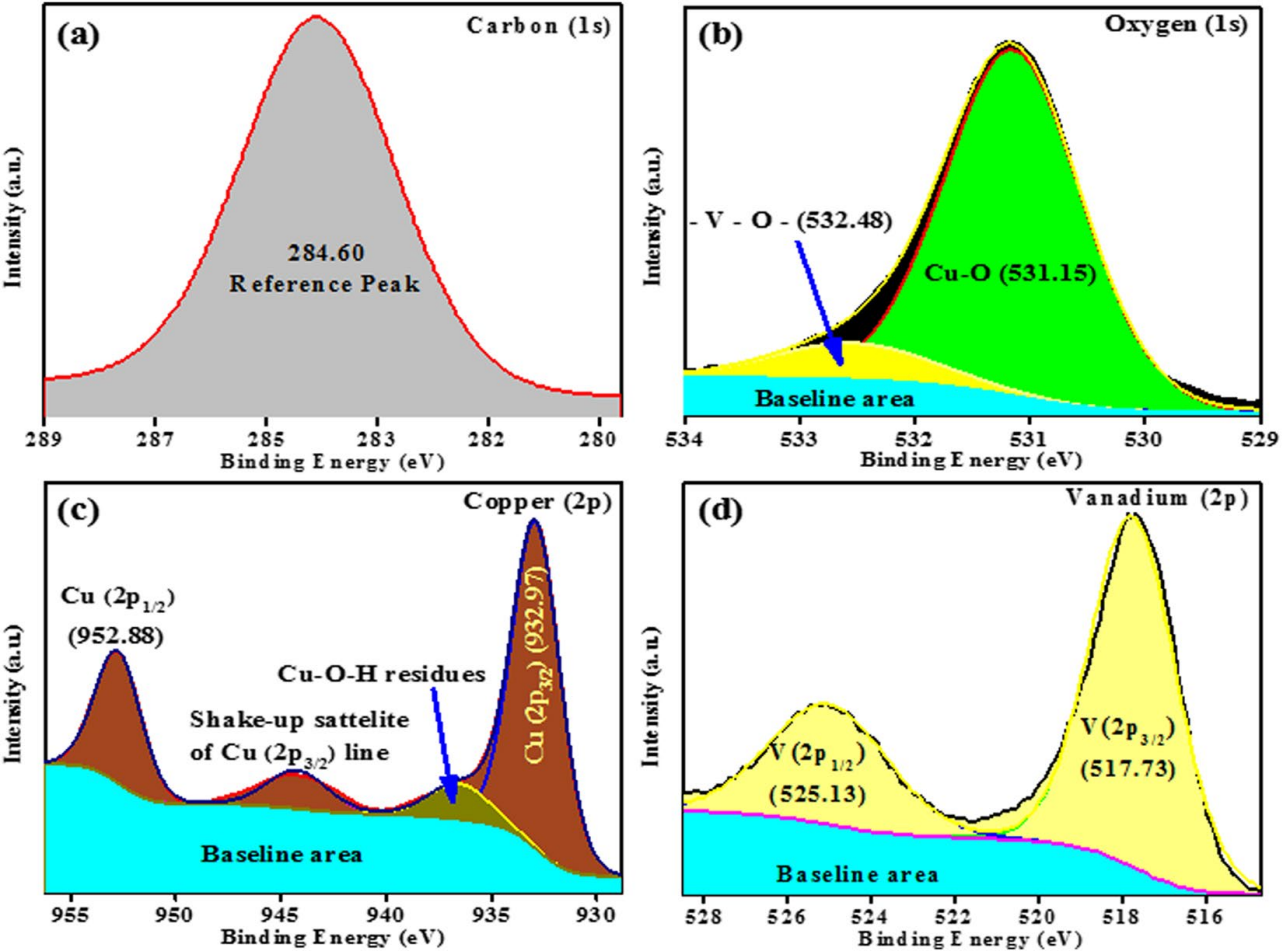
Deconvoluted XPS profiles of constituent elements of CV-500.

To further investigate the detailed composition and chemical states of the constituent elements of CV-500 sample, XPS was carried out (Fig. 5a–d). The high-resolution XPS profile survey obtained after 30 s CV-500 film surface etching is provided in supporting information (Fig. S1). The magnified deconvoluted portions of the survey are shown in Fig. 5. Figure 5(a) represents the characteristic XPS corrected peaks for carbon at 284.6 eV, which is used as a reference to obtain the exact peaks values. The fitted oxygen peak in Fig. 5(b) shows two different deconvoluted regions. These asymmetric peaks could be associated with the bonding of oxygen with vanadium and copper in the final structure as revealed from the literature^30^. The strong deconvoluted peak, which is centered at 532.15 eV is previously reported for the Cu-O bond. Similarly, the centered weak peak at 532.48 eV is reported for most vanadates, and assigned to V-O bonding. The major peaks for V(2p) and Cu(2p) found in the survey spectrum as well. Two peaks V2p_3/2_ (517.73 eV) and V2p_1/2_ (525.13 eV) can be indexed to V^5+^ according to the available literature^31,32^. Copper shows strong Cu^2+^ oxidation state in CV-500, as the characteristic doublet with shake-up satellite peak is observed at 932.97 (Cu 2p_3/2_), 944.38 (shake-up satellite of Cu 2p_3/2_) and 952.88 (Cu 2p_1/2_) eV, respectively. The shake-up satellite is the signature peak of Cu (II) oxidation state^18,25,33,34^. These values of binding energies are consistent with the previously reported results. These results confirm that CV-500 contains Cu^2+^, V^4+^, and O^2−^. The empirical formula for the combination is achieved to be CuV_2_O_6_, which is consistent with the XRD results.

**Figure 6:**
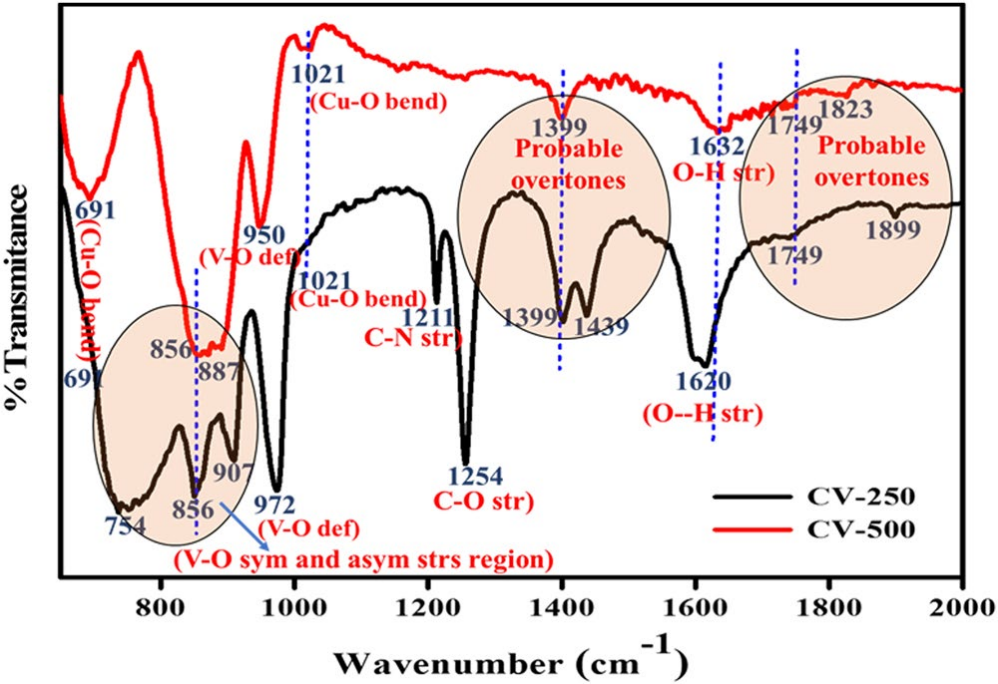
FT-IR spectroscopy spectra of CV-250 and CV-500 nanoparticles.

Figure 6 shows FT-IR spectra of the CV-250 and CV-500 samples, collectively from the range of 600 cm^−1^ to 2000 cm^−1^, where most of the metal oxide species show significant vibrational characteristics^35^. For the CV-250 Peaks at 1439, 1254 and 1211 assigned to C-C and C-N stretches. These peaks completely disappear in CV-550, which indicated the presence of impurities from *P-123* surfactant in the CV-250 sample. The infrared band at 1620 correspond to CV-250 and 1632 cm^−1^ in CV-550 assigned to the O-H bending vibration of water molecules^26^. The intensity of this band significantly decreases in the later case, contributed to their high purity and single phase crystallinity. Most of the bands above 1100 caused due to overtones. For example, the band at 1021 is attributed to Cu–O deformation vibrations, while, the bands observed at 1399, 1749, 1829 and 1899 are caused by probable overtone and/or combination bands^36^. The bands in encircled region from 750 to 910 cm^−1^ relate to the V–O symmetric and asymmetric stretching vibrations. Finally, the band at 691 cm^−1^ assigned to Cu–O bending.

**Figure 7:**
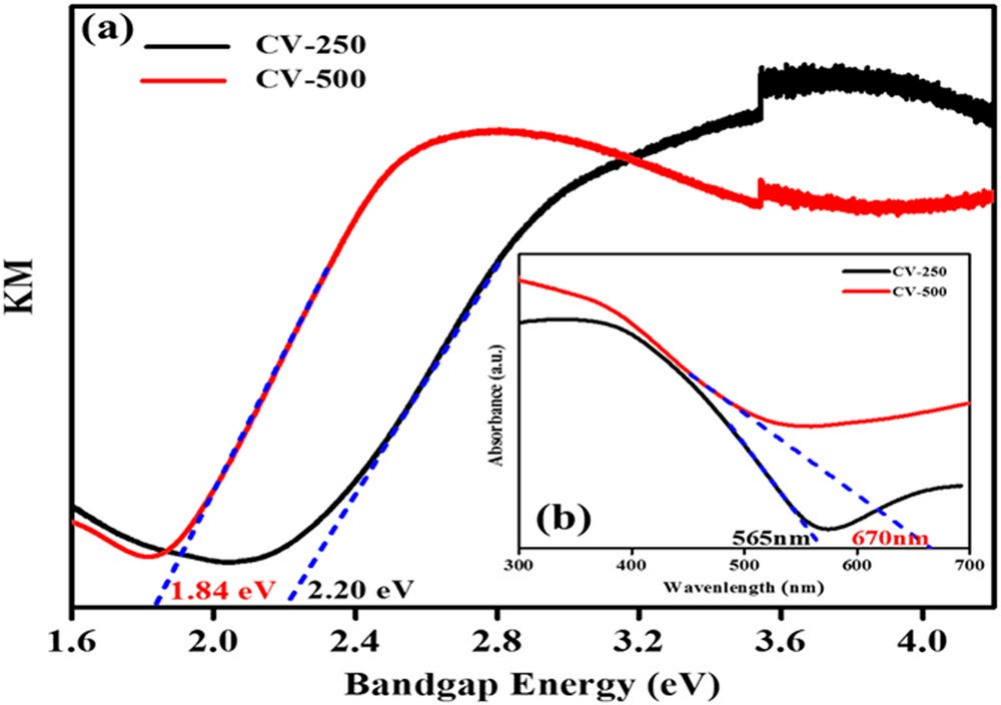
Optical characteristics of CV-250 and CV-550: (**a**) Tauc’s plots and (**b**) absorption spectra (inset), for band gap and optical band edge determination.

Figure 7 represent the optical properties of the CV-250 and CV-500 samples in terms of Tauc’s plots (Fig. 7a), which was obtained from UV-DRS. Both sample show significantly lower band gap of 2.2 eV and 1.84 eV, respectively. The absorption is substantially enhanced in the case of CV-500 as shown in the inset (Fig. 7b). The optical absorption band edges of CV-250 and CV-500 observed at 565 and 670 nm, respectively. The enhancement of absorption in the case of CV-500 can be attributed to the small crystal size, which directly increases the surface area, and hence provide more space for visible light absorption. The optical properties of CV-250 and CV-500 seem suitable for PEC water splitting reaction as the band gap benchmark value for this reaction is 1.5–2.5 eV.

Optical band edge position calculations of CV-250 and CV-550 ware performed by using Tauc’s plot (band gaps values) to reveal the liability of these materials towards water splitting reaction. As anticipated, the band gap (E_g_) is the energy difference valence band (E_CB_) and conduction band (E_CB_). Therefore,

1 $${{\bf{E}}}_{{\bf{g}}}={{\bf{E}}}_{{\bf{VB}}}\mbox{--}{{\bf{E}}}_{{\bf{CB}}}$$

Theoretically, equation (1) can be used to calculate the value of any parameter by knowing the value of other two parameters. Tauc’s plot gave the E_g_ value, while the E_CB_ can be determined by equation (2).

2 $${{\bf{E}}}_{{\bf{CB}}}={\boldsymbol{\chi }}\mbox{--}{{\bf{E}}}_{{\bf{C}}}\mbox{--}{\boldsymbol{1/2}}{{\bf{E}}}_{{\bf{g}}}$$

where, E_C_ is the energy of free electron i.e. 4.5 eV and **χ** is Mullikan’s absolute electronegativity, which can be calculated by taking the sum of the arithmetic mean of the atomic electron affinity and the first ionization energy of the component elements (equation 3).

3 $${\boldsymbol{\chi }}={\boldsymbol{1/2}}\,({\bf{Eelectronaffinity}}+{{{\bf{E}}}_{{\bf{1}}}}^{{\bf{st}}}{\bf{ionization}}\,\,{\bf{energy}})$$

**Figure 8:**
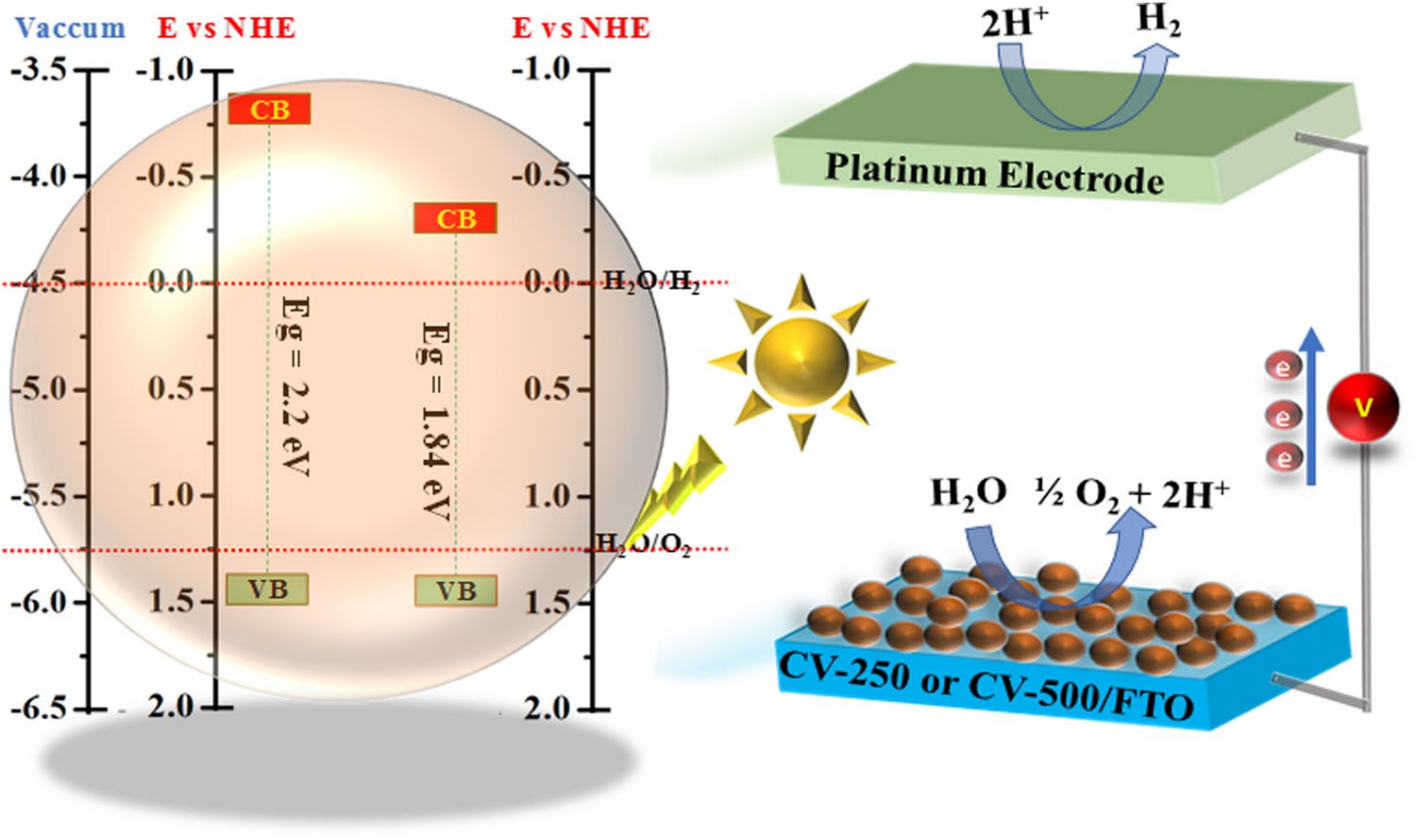
Band edge positions and PEC water splitting mechanism of CV-250 and CV-500.

The **χ** values for CV-250 and CV-500 samples calculated to be 6.02 eV and 6.05 eV and after putting these values in equation (2), we get −0.68 eV and −0.28 eV E_CB_ values for both the samples, respectively. Similarly, the E_VB_ band edge values for CV-250 and CV-500 is measured from equation (1) by inserting the values of E_g_ and E_CB_ and the final values were 1.52 eV and 1.55 eV. Figure 8 shows the calculated band edge positions of CV-250 and CV-500 with water splitting mechanism.

The growth mechanism of CV-500 can be predicted based on the available literature. Different from the previous reports, *P-123* has been used as a surfactant in the present experimental protocol. *P-123* selectively produced platelet-shaped morphology with nanoscale size at 500C°. The controlled size and shape syntheses have been achieved via controlling the pH of the hydrothermal solution, and specific reaction and calcination temperature. The role of time is also investigated and the minimum hydrothermal reaction time was determined to be 12 hrs at 120 °C. Additionally, the concentration of *P-123* also effect the product formation and therefore after thorough literate review, the optimum amount of the surfactant has been used. It is clear that the existence of hydrogen ions or hydroxyl ions accelerates the formation of the copper vanadate nanosheets^19^. On the basis of the experimental results, it is supposed that the formation of the nanoplate-shaped CV-500 NPs can be ascribed to the CV-120 and CV-250 nanosheets. The growth process of the CV-500 has been anticipated based on the nucleation and *P-123* adsorption growth mechanism normal pH conditions (pH = 7.3). Figure 1 demonstrates the schematic of the *P-123*-assisted growth of the platelet-shaped CV-500 NPs. *P-123* belongs to a class of non-ion surfactants and offers coordination positions/sites, which contain necessary heterogeneous nucleation sites. At the earliest reaction phase, enough energy provided to the precursors through pulse probe sonication to make their ions i.e. Cu, V, O etc. Additionally, sonication also assists the homogeneous dispersion of these ions in the solution medium. In the second phase, this reaction mixture was transferred to an autoclave for further treatment. *P-123* act as ions trapper and form uniform micelles, each micelle act as a small reaction chamber. During this period, the ions form seeds of copper vanadate, which grow and probably form clusters. In the final phase, the product is kept for calcination for 12 h at 250 and 600, respectively. The sand flower shaped sheets are formed with more distinct boundaries in case the of CV-250 with the disappearance of most of *P-123*. Further temperature completely digests *P-123*, and the platelet-shaped CV-550 NPs obtained with high-purity and crystallinity.

**Figure 9:**
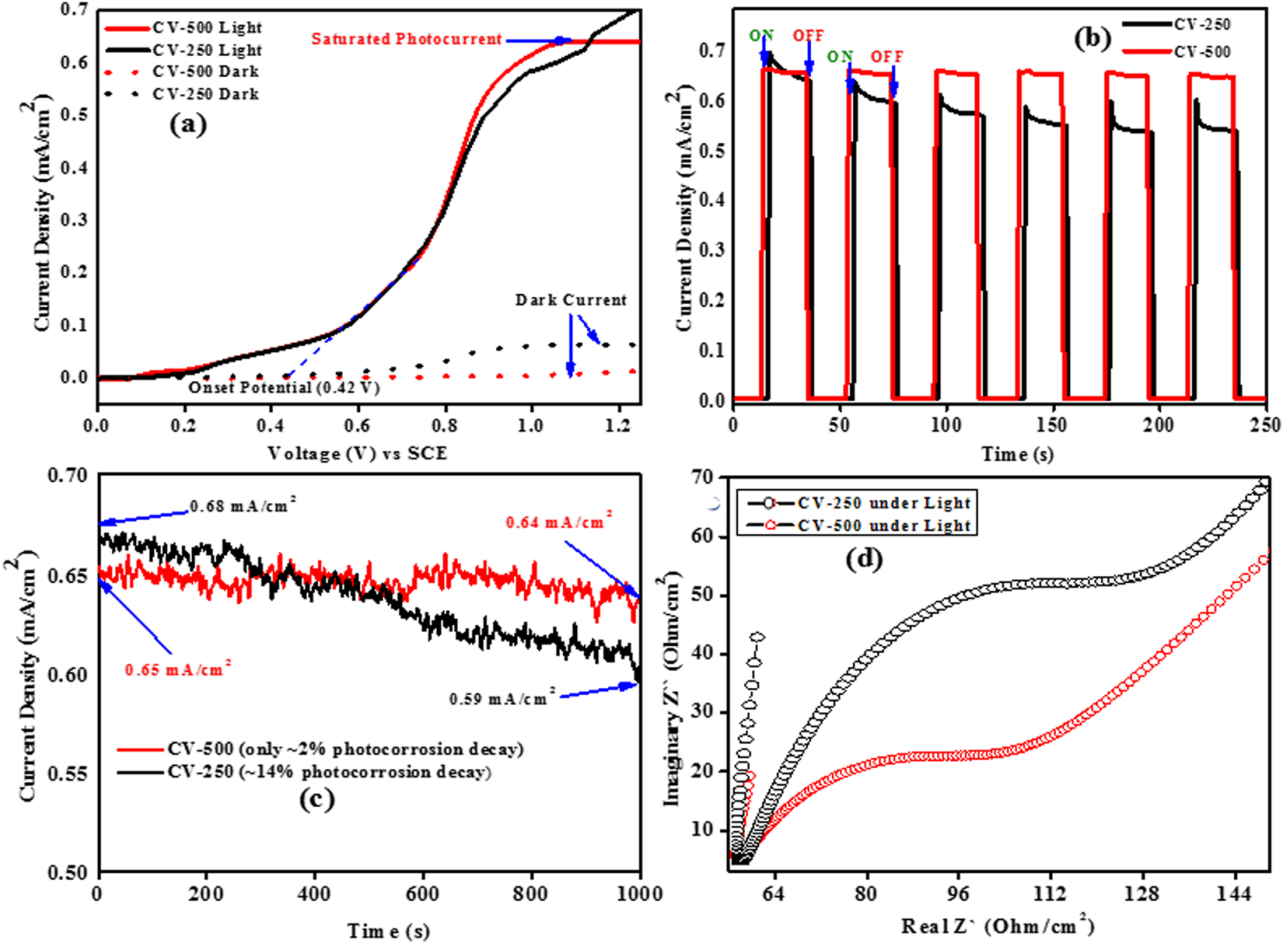
Photoelectrochemical measurements of CV-250 and CV-500 NPs: (**a**) linear-sweep voltammograms *(I-V)*, (**b**) Photocurrent under periodic ON/OFF solar irradiations, (**c**) chronoamperometric *(I-t)* stability curve, and (**d**) electrochemical impedance spectroscopy (EIS) spectra under light.

The PEC performance of the CV-250 and CV-500 photoelectrodes was explored via linear-sweep voltammetry (LSV) and chronoamperometry. Figure 9(a) presents the LSV measurements under regular ON/OFF solar illumination. In all cases, the dark current remained significantly lower, while under the simulated light up to 1.2 V, the photocurrent densities increased enormously with the voltage sweep, which is indicated by the *I-V* spectra. The results revealed that the photocurrent density is same almost same for both materials. The *I-V* spectra also revealed the onset point of photocurrent at 0.4 V, and the plateau is observed due to photo current saturation after 0.9 V, which remains stable until 1.2 V. The current beyond 1.2 V showed high jump, which indicated that electrochemical processes dominated and the contribution of photocurrent became insignificant after this range. The photocurrent densities observed at the thermodynamic potential (1.23 V) of the water oxidation reaction were ~0.70 and ~0.64 mA/cm^2^ for BV-250 and BV-500_2,_ respectively. A significant dark current appeared above 1.23 V for the BV-250 and BV-500 photoelectrodes, which can be attributed to electrochemical processes^37^. The chronoamperometric (*I-t*) results collected under successive light and dark cycles with ~20 s intervals further supported the results (Fig. 9b). The *I-t* ON/OFF cycles indicated that during light chopping, the photocurrent reached a maximum value under illumination and diminished in the dark at 1.23 V. The photocurrent values under the light are almost of the same order as those obtained using LSV.

Figure 9(c) shows the long-term *I-t* stability curve obtained from photoanodes under light with an illumination period of 1000 s. For the BV-500 photoelectrode, the stability curve insignificantly decreased from ~0.65 to ~0.64 mA/cm^2^ and show only ~2% photocorrosion decay during this period. For BV-250, the stability curve decreased significantly over the time, with approximately 14% photo-decay, which can be attributed to the PEC corrosion of this material. As expected, BV-500 showed considerable resilience and stability for most of the period.

Electronic impedance spectroscopy (EIS) was performed to investigate the interfacial charge transfer in BV-250 and BV-500 photoelectrodes from the electrolyte to the surface of the electrode (point of reaction). All samples were analyzed under simulated solar illumination with a DC voltage sweep across the working electrode. Nyquist plot spectra with representative semicircles are provided in Fig. 9(d) for both photoelectrodes. The CV-500 showed exact semicircular Nyquist plot with relatively lower resistance than CV-250^38^. The lower real and imaginary resistant of CV-500 suggested that it provided a good interface for charge transfer. It can also have proposed that the charge recombination in the case of CV-500 is minimized due to which it shows relatively good stability than CV-250.

**Figure 10:**
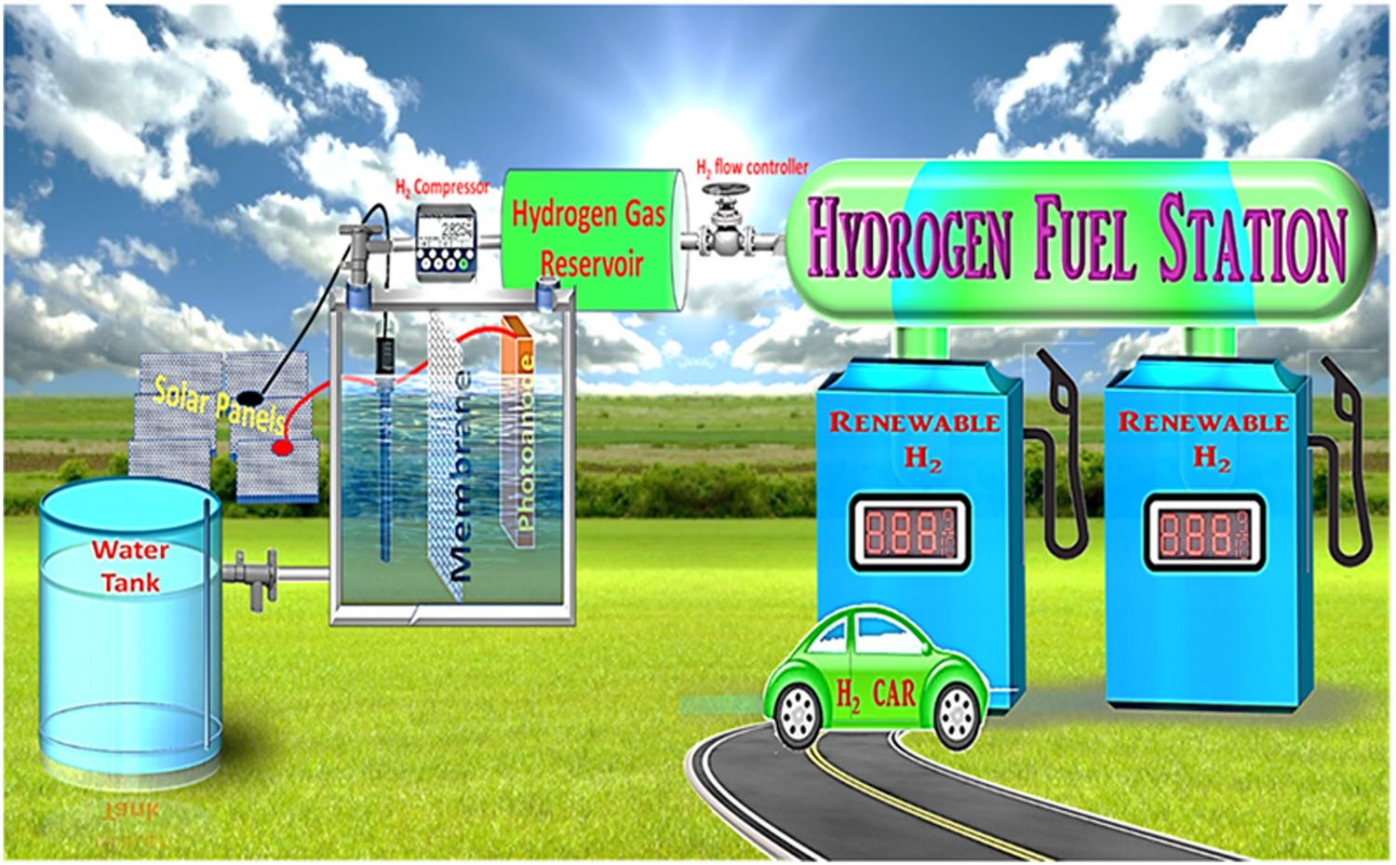
Scheme An overview of clean energy harvesting through PEC water splitting under sun light.

The photocurrent densities achieved in this study are significant compared with those in the literature^39–41^. Table 1 provides comparative current densities obtained from the copper vanadate nanostructures. The reported photocurrent values indicate that our material generates comparable photocurrent density, in addition to the facile sol gel method used, which is less expensive and can produce large scale product. Additionally, the shape and size control synthesis of copper vanadate demonstrated good photocatalytic behavior than usual. A general schematic for clean energy hydrogen from sun light and its potential application as a clean fuel is depicted in Fig. 10.

Based on the information from optical properties, band edge position, and impedance electron microscopy, the possible mechanism for photocurrent generation can be proposed for CV-250 and CV-500^42,43^. The band gaps of CV-250 and CV-500 are 2.2 eV and 1.84 eV, respectively from the Tauc’s plot. This is the appropriate range of band gap, where the material can efficiently split water with the help of solar energy. Additionally, the band edge positions of CV-250 and CV-500 straddle the redox potentials for water photolysis as predicted from Fig. 8. Thus, it can be assumed that these materials are suitable for overall PEC water splitting due to the alignment of the band edges with the redox potential of water i.e. 0.0 V and 1.23 V. CV-250 and CV-500 absorb light in the visible regime, which excited the electrons from the E_VB_ of CV-250 and CV-500 to E_CB_ and produced e^−^/h^+^ pairs. The life time of e^−^/h^+^ pairs is very important to increase the efficiency of the photoelectrode material as the lower recombination rate is always favorable for effective photocatalytic applications^43,44^. The holes are readily available for oxygen evolution reaction (OER) at the photoelectrode surface and electrons induced hydrogen evolution reaction (HER) at the surface of the counter electrode. In the case of CV-500, the electrode showed much stability over the given period as compared to CV-250. This stability can be attributed to the lower e^−^/h^+^ pair recombination as revealed from the EIS spectra. The overall results and proposed mechanism indicated that copper vanadate can be used as an effective photo-material for water splitting after controlling the stability and lowering the e^−^/h^+^ pairs recombination via surface modification, doping, heterostructure formation with other materials and/or cocatalysts.

In summary, we effectively performed shape controlled synthesis of a triclinic form of copper vanadate and tested their PEC water splitting performance for the first time to best of our knowledge. FESEM revealed the distinct semi spherical platelets shaped nano particles of CV-550. The XRD established the existence of triclinic crystal phase of CV-500. Moreover, EDX and elemental mapping confirmed the presence of Cu, V and O elements. The DRS spectra designated the absorption in the visible region, while the Tauc’s plots reveal the bandgap of 1.84 eV for CV-500. The band edge positions of CV-250 and CV-500 straddle the redox potential of water, suggesting that they are suitable for overall water splitting. The PEC results indicated the highest current density for the CV-250 and CV-500 is 0.70 mA/cm^2^ and 0.68 mA/cm^2^, respectively. However, the former is unstable and show immense photocurrent dip with the time as revealed from the stability curve. The EIS of CV-250 and CV-500 comprehend that in addition to lower pair recombination, the interfacial charge transfer is also greater in the case of CV-500. CV-500 can be an efficient addition to photoelectrode material for PEC water splitting applications, owing to suitable optoelectrical characteristic. The stability and efficiency of this materials can be control by their surface modification, doping, cocatalysis and other reliable techniques.

## Methods

### Synthesis of CV-120, CV-250 and CV-500 nanoparticles

In the simple experiment, the calculated amount of Cu(CO_2_CH_3_)_2_ and NH_4_VO_3_ was taken in the respective beakers in 25 ml ethanol solvent. The precursors quantity was adjusted at 1:2 by mass of vanadium (V) and copper (Cu), respectively. Both solutions were mixed by through stirring before transferring into one beaker. The calculated amount of *P-123* also added to the mixture, whose amount was 10 times greater than NH_4_VO_3_ by mass %. The large mass of *P-123* is necessary to form micelles in the reaction mixture, which provide small reaction chambers for the formation of nanostructures. The mixture further subjected to pulse sonication for 30 min. through ultra/probe Sonicator (SONICS-4000 Vibra-Cell) operated at 20 amperes at 50 °C. The pulses were provided at 10 s ON and 3 s OFF cycles. After ultrasonic probe treatment, the solution was transferred to sealed Teflon lined autoclave and kept at 120 °C for 12 h. Finally, the sample was washed to remove unreacted ions dried and divided into three portions. Each portion is kept at different calcination temperature i.e. 120 °C, 250 °C and 500^o^C, respectively. The final products were labeled as CV-120 (Blackish color), CV-250 (dark greenish/yellow color) and CV-500 (dark reddish/brown) owing to their calcination temperature. Complete pathway of the reaction is provided in Fig. 1.

### Materials and Physical Measurements

Laboratory grade precursors copper(II) acetate (Cu(CO_2_CH_3_)_2_) and ammonium metavanadate (NH_4_VO_3_) along with symmetric triblock copolymer Pluronic *P-123* [poly(ethylene glycol)-poly (propylene glycol)- poly(ethylene glycol)] surfactant were purchased from Sigma Aldrich and used as received. For characterization of the products, the morphological topography, shape, and size were explored using a field emission scanning electron microscope (FESEM). SEM micrographs were obtained using a LYRA 3 Dual Beam instrument (Tescan) operated at an acceleration voltage of 20 kV. This instrument was also equipped with an energy dispersion spectrometer (EDX, Oxford Instruments), which was used to determine the elemental ratio and confirm the constituent elements and mapping. X-ray characterization was performed using a Rigaku X-ray diffractometer equipped with a Cu Kά (λ = 0.15406 nm) radiation source and operated from 2θ = 20° to 70° at a scan rate of 2 degree/min. to determine the facet arrangement and crystalline phase of the CV-250 and CV-500 heterostructures. Fourier transform infrared (FT-IR) spectra from 600 to 2000 cm^−1^ were recorded on Thermo-Scientific FT-IR instruments. An ESCALAB 250Xi X-ray photoelectron spectrometer (XPS) microprobe device was utilized to study the oxidation details of the component elements in the CV-500. A Carry 5000 model was used to investigate optical properties of the samples. Absorption spectra were recorded from 200–800 nm at a scan rate of 100 nm/min. In addition, Tauc’s plots were recorded from 1.5 to 4 eV to calculate the exact band gap using the Kubelka-Munk (KM) equation^8^.

### Photoelectrode fabrication and Photoelectrochemical measurements

PEC water splitting was performed in a three-electrode PEC cell containing a 0.5 M Na_2_SO_4_ (Sigma Aldrich) electrolyte (pH 7.2). The CV-250 and CV-500 deposited FTOs were used as the working electrodes and were controlled by an Autolab potentiostat. Platinum (Pt) and saturated calomel electrode (SCE) served as the counter and reference electrodes, respectively. All PEC measurements were recorded using NOVA software installed on a data collecting device under the controlled chopping of dark and light. A solar simulator (Oriel Sol-3A Newport) provided artificial solar light irradiation, and the power was calibrated using a silicon diode solar cell (Oriel-diode) and fixed at 100 mW/cm^2^, which is equivalent to 1 SUN. The solar simulator was also equipped with AM-1.5 G and UV cutoff (λ > 420 nm) filters.

## Electronic supplementary material

Supplementary Information
