# Supplementary material for: Shape Controlled Synthesis of Copper Vanadate Platelet Nanostructures, Their Optical Band Edges, and Solar-Driven Water Splitting Properties
Source: Sci Rep. 2017 Oct 30;7:14370. doi: 10.1038/s41598-017-14111-7 (PMC5662690; doi:10.1038/s41598-017-14111-7)
Supplement: Supplementary file 1 — Supplementary Information [file 41598_2017_14111_MOESM1_ESM.pdf]

Supplementary Materials

Shaped Controlled Synthesis of Copper Vanadate Platelet Nanostructures, Their Optical Band Edges and Solar-Driven Water Splitting Properties

Ibrahim Khan[1,2], *Ahsanulhaq Qurashi*[1,2*]

[1]Center of Research Excellence in Nanotechnology King Fahd University of Petroleum and Minerals, Dhahran, Saudi Arabia

[2]Chemistry Department, King Fahd University of Petroleum and Minerals, Dhahran, Saudi Arabia

Figure S1 represents the FESEM images of copper vanadate samples at 150 ^{o}C (a-c) and 200 ^{o}C (d-f). The calcination temperature gradually changes the morphology from non-regular to somewhat regular shape.

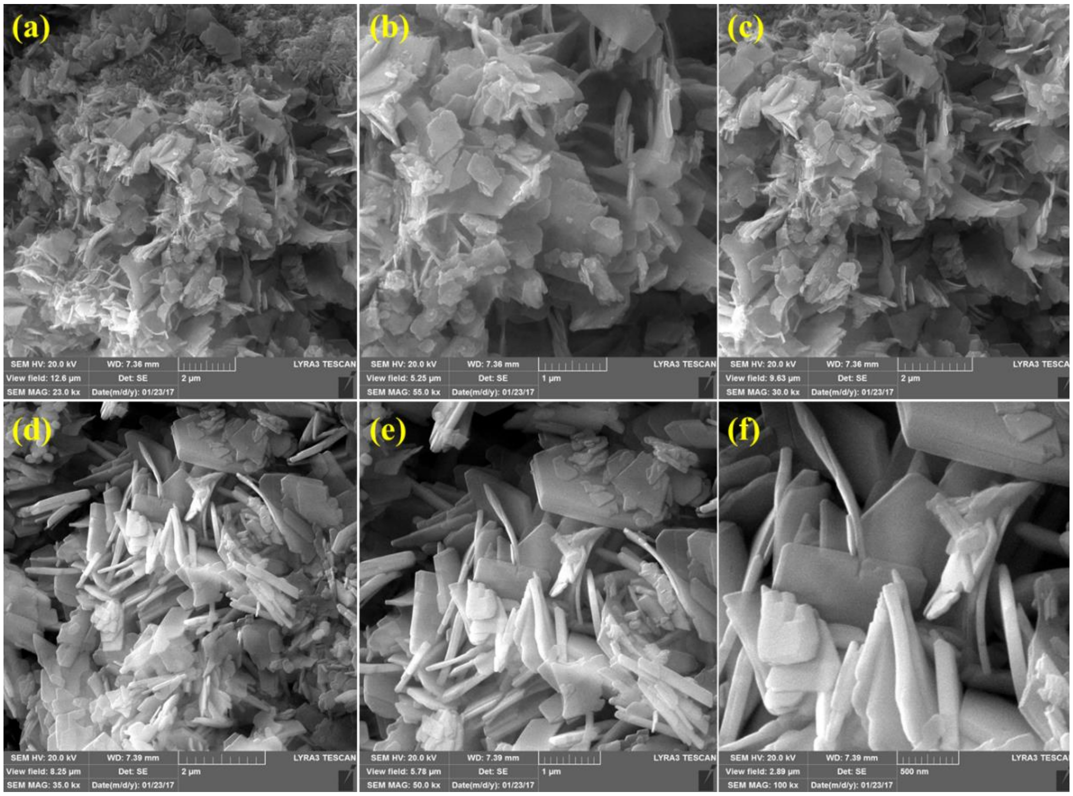

Figure S1. FESEM images of copper vanadate calcined at 150 ^{o}C (a-c) and 200 ^{o}C (d-f).

Figure S2 represents the XPS profiling of CV-500 Photoanode film (took after 30s etching). The profile contains all characteristic XPS peaks of CuV2O6, which are discussed in the discussion section of the manuscript in details.

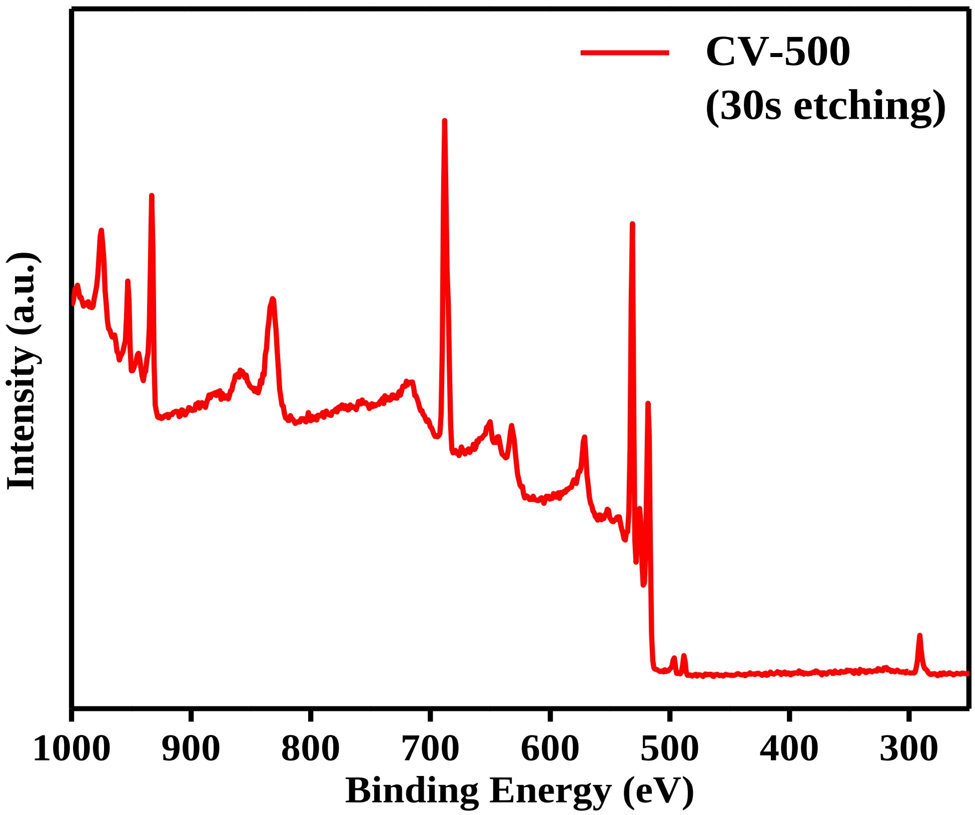

Figure S1. XPS Survey for CV-500 Photoanode film (took after 30s etching)

Table S1. XRD Parameters of CV-500 and CV-250

**CV-500**				**CV-250**			
2-theta (deg)	d(ang.)	Size	Phase data name	2-theta (deg)	d(ang.)	Size	Phase data name
14.61	6.06	539.67	(0 0 1)	18.73	4.73	572.95	(0 0 2)
20.70	4.29	559.42	(2 0 0)	21.04	4.22	583.16	(1 1 1)
20.77	4.27	559.60	(2 0 -1)	21.60	4.11	605.71	(1 1 -2)
26.80	3.32	572.00	(1 -1 0)	22.16	4.01	611.26	(0 2 0)
27.77	3.21	573.21	(1 1 0)	24.10	3.69	621.33	(0 2 1)
29.02	3.07	574.41	(1 1 -1)	24.71	3.60	633.11	(2 0 0)
29.40	3.04	574.69	(2 0 1)	25.35	3.51	646.82	(2 0 -2)
29.47	3.03	574.74	(0 0 2)	28.17	3.17	649.62	(1 1 2)
29.52	3.02	574.77	(1 -1 -1)	28.89	3.09	654.13	(1 1 -3)
29.55	3.02	574.79	(2 0 -2)	29.18	3.06	667.15	(0 2 2)
31.76	2.82	575.63	(1 -1 1)	32.28	2.77	668.05	(2 2 -1)
33.85	2.65	575.26	(1 1 1)	33.43	2.68	689.84	(2 2 0)
36.93	2.43	572.65	(1 1 -2)	33.92	2.64	572.95	(2 2 -2)
38.45	2.34	570.48	(1 -1 -2)	35.82	2.50	583.16	(1 3 0)
38.66	2.33	570.13	(3 -1 -1)	35.94	2.50	605.71	(1 3 -1)
39.35	2.29	568.94	(4 0 -1)	37.35	2.41	611.26	(3 1 -2)
39.53	2.28	568.60	(3 -1 0)	37.54	2.39	621.33	(2 0 -4)
39.69	2.27	568.30	(3 1 -1)	43.30	2.09	633.11	(1 3 -3)
41.59	2.17	564.30	(3 1 0)	43.44	2.08	646.82	(3 1 1)
41.98	2.15	563.36	(1 -1 2)	44.02	2.06	649.62	(2 2 -4)
42.08	2.15	563.14	(2 0 2)	44.43	2.04	654.13	(0 2 4)
42.12	2.14	563.05	(4 0 0)	44.91	2.02	667.15	(3 1 -4)
42.25	2.14	562.71	(2 0 -3)	45.22	2.00	668.05	(0 4 0)
42.26	2.14	562.70	(4 0 -2)	45.69	1.98	689.84	(1 1 4)
43.42	2.08	559.71	(3 1 -2)	46.28	1.96	704.04	(0 4 1)
43.47	2.08	559.55	(3 -1 -2)	46.55	1.95	716.84	(1 1 -5)
44.64	2.03	556.27	(1 1 2)	47.37	1.92	572.95	(4 0 -2)
44.85	2.02	555.65	(0 0 3)	49.68	1.83	646.82	(3 3 -2)
45.81	1.98	552.72	(3 -1 1)	49.98	1.82	649.62	(2 2 3)
48.57	1.87	543.46	(3 1 1)	50.67	1.80	563.05	(4 0 0)
48.73	1.87	542.90	(1 1 -3)	55.53	1.65	562.71	(2 4 -3)
49.79	1.83	539.01	(4 0 1)	55.91	1.64	562.70	(3 3 -4)

50.04	1.82	538.08	(4 0 -3)	55.95	1.64	559.71	(4 2 0)
50.84	1.79	535.01	(1 -1 -3)	56.35	1.63	559.55	(1 1 -6)
51.73	1.77	531.56	(0 2 0)	56.59	1.63	556.27	(1 3 4)
51.79	1.76	531.31	(3 1 -3)	56.78	1.62	555.65	(3 1 3)
52.71	1.74	527.63	(3 -1 -3)	57.26	1.61	552.72	(4 2 -4)
53.20	1.72	525.64	(0 2 -1)	57.32	1.61	543.46	(1 3 -5)
54.89	1.67	518.56	(0 2 1)	58.25	1.58	542.90	(2 2 4)
55.13	1.66	517.55	(1 -1 3)	58.98	1.56	535.01	(1 5 0)
55.22	1.66	517.17	(2 -2 0)	59.06	1.56	531.56	(1 5 -1)
55.78	1.65	514.77	(5 -1 -1)	59.22	1.56	531.31	(2 4 2)
56.07	1.64	513.53	(3 -1 2)	59.84	1.54	527.63	(3 3 2)
56.08	1.64	513.48	(2 -2 -1)	59.84	1.54	525.64	(2 2 -6)
56.57	1.63	511.37	(2 2 -1)	60.10	1.54	518.56	(4 2 1)
56.73	1.62	510.70	(2 0 3)	60.17	1.54	517.55	(2 4 -4)
56.93	1.62	509.83	(2 0 -4)	60.50	1.53	517.17	(0 4 4)
57.36	1.60	507.92	(2 2 0)	60.66	1.53	514.77	(1 5 1)
57.38	1.60	507.85	(5 -1 -2)	60.90	1.52	513.53	(1 5 -2)
57.63	1.60	506.76	(5 1 -1)	61.03	1.52	513.48	(4 0 2)
58.14	1.59	504.52	(1 1 3)	61.48	1.51	511.37	(3 3 -5)
58.38	1.58	503.42	(5 1 -2)	61.51	1.51	510.70	(5 1 -2)
58.61	1.57	502.41	(5 -1 0)	61.90	1.50	509.83	(5 1 -3)
58.79	1.57	501.61	(2 -2 1)	61.97	1.50	507.92	(4 2 -5)
59.07	1.56	500.39	(0 2 -2)	62.84	1.48	507.85	(5 1 -1)
59.28	1.56	499.44	(3 1 2)	63.30	1.47	509.83	(0 2 6)
60.15	1.54	495.57	(2 2 -2)	63.50	1.46	507.92	(4 0 -6)
61.00	1.52	491.71	(4 0 2)	63.99	1.45	507.85	(5 1 -4)
61.14	1.51	491.07	(0 0 4)	64.01	1.45	506.76	(1 5 2)
61.15	1.51	491.04	(6 0 -1)	64.39	1.45	504.52	(1 5 -3)
61.18	1.51	490.89	(5 1 0)	70.23	1.34	511.68	(4 4 0)
61.23	1.51	490.67	(6 0 -2)	70.37	1.34	515.94	(5 1 1)
61.26	1.51	490.57	(2 -2 -2)	70.43	1.34	517.64	(0 6 0)
61.33	1.51	490.24	(4 0 -4)	70.60	1.33	522.76	(3 5 0)
62.22	1.49	486.22	(0 2 2)	70.78	1.33	528.55	(5 3 -2)
62.41	1.49	485.34	(2 2 1)	71.14	1.32	540.46	(5 3 -3)
62.92	1.48	483.06	(1 1 -4)	71.23	1.32	543.56	(0 6 1)
63.18	1.47	481.88	(5 -1 -3)	71.25	1.32	544.07	(3 5 -3)
63.36	1.47	481.05	(5 1 -3)	71.39	1.32	548.92	(4 4 -4)
63.51	1.46	480.39	(3 1 -4)	72.02	1.31	572.95	(5 3 -1)

65.08	1.43	473.29	(3 -1 -4)	72.27	1.31	583.16	(2 4 4)
65.23	1.43	472.62	(6 0 0)	72.78	1.30	605.71	(4 2 3)
65.46	1.42	471.55	(6 0 -3)	72.89	1.30	611.26	(5 1 -6)
65.48	1.42	471.48	(1 -1 -4)	73.10	1.29	621.33	(5 3 -4)
65.50	1.42	471.38	(5 -1 1)	73.32	1.29	633.11	(2 0 6)
65.68	1.42	470.56	(4 -2 -1)	73.57	1.29	646.82	(3 5 1)
66.35	1.41	467.55	(2 -2 2)	73.62	1.29	649.62	(0 6 2)
68.06	1.38	459.85	(4 2 -1)	73.93	1.28	668.05	(4 4 1)
68.49	1.37	457.93	(4 -2 -2)	74.26	1.28	689.84	(3 3 4)
68.63	1.37	457.30	(0 2 -3)	74.47	1.27	704.04	(6 0 -2)
68.66	1.37	457.16	(5 1 1)	74.64	1.27	716.84	(3 5 -4)
69.23	1.36	454.63	(3 -1 3)	74.72	1.27	722.78	(3 1 5)
69.36	1.35	454.05	(4 2 -2)	74.83	1.27	731.92	(1 3 6)
70.18	1.34	450.45	(2 -2 -3)	74.83	1.27	732.19	(5 3 0)
70.41	1.34	449.43	(1 -1 4)	75.22	1.26	765.46	(1 5 4)
70.72	1.33	448.03	(4 2 0)	75.27	1.26	770.15	(2 6 -1)
71.21	1.32	445.90	(2 2 2)	75.31	1.26	774.13	(2 0 -8)
72.05	1.31	442.20	(4 -2 1)	75.32	1.26	774.56	(6 0 -4)
72.07	1.31	442.12	(5 1 -4)	75.62	1.26	805.04	(4 2 -7)
72.62	1.30	439.74	(5 -1 -4)	75.63	1.26	806.32	(4 4 -5)
73.09	1.29	437.74	(6 0 1)	75.91	1.25	838.30	(2 6 0)
73.12	1.29	437.59	(2 0 4)	76.19	1.25	874.23	(2 6 -2)
73.34	1.29	436.62	(2 0 -5)	76.36	1.25	897.17	(5 1 2)
73.46	1.29	436.13	(6 0 -4)	76.76	1.24	962.44	(4 0 4)
73.76	1.28	434.82	(1 1 4)	76.85	1.24	978.50	(0 4 6)
74.54	1.27	431.52	(4 2 -3)	76.99	1.24	1005.7	(1 1 7)
74.98	1.27	429.66	(4 0 3)	77.46	1.23	1119.7	(3 1 -8)
75.12	1.26	429.05	(4 -2 -3)	77.54	1.23	1142.6	(0 6 3)
75.37	1.26	428.00	(4 0 -5)	77.70	1.23	1192.30	(2 2 6)
75.88	1.25	425.84	(5 -1 2)	78.04	1.22	1324.90	(1 1 -8)
77.09	1.24	420.82	(7 -1 -2)	78.06	1.22	1336.60	(3 5 2)
77.18	1.23	420.47	(4 2 1)	78.11	1.22	1360.20	(2 6 1)
77.32	1.23	419.90	(2 -2 3)	78.46	1.22	1580.50	(6 2 -3)
77.60	1.23	418.72	(7 -1 -1)	78.67	1.22	1766.70	(2 6 -3)
77.94	1.22	417.33	(3 1 -5)	78.82	1.21	1959.40	(6 2 -2)
78.60	1.22	414.63	(2 2 -4)	79.11	1.21	2625.40	(4 4 2)
78.87	1.21	413.54	(7 1 -2)	79.16	1.21	2795.30	(5 3 1)
